# Editorial: Economic and social factors affecting the health of older adults

**DOI:** 10.3389/fpubh.2023.1323030

**Published:** 2023-11-22

**Authors:** Colette J. Browning, Katherine H. Leith, Shane A. Thomas

**Affiliations:** ^1^Institute of Health and Wellbeing, Federation University Australia, Ballarat, VIC, Australia; ^2^Research School of Population Health, Australian National University, Canberra, ACT, Australia; ^3^College of Social Work, University of South Carolina, Columbia, SC, United States; ^4^Vice Chancellor's Office, Federation University, Ballarat, VIC, Australia

**Keywords:** aging, economic factors, social factors, healthy aging, Research Topic, global health

The world is undergoing a period of global population aging; in 2020, over 1 billion of the world's population was over 60 years old, and this number is expected to double by 2050 (1). The UN has promoted the UN Decade of Healthy Aging to transform the lives of older people by “adding life to years” and reducing health inequalities (2). Healthy aging policies are responses to the potential to optimize health and wellbeing as we age and to challenge the “burden of aging” rhetoric (1). Such policies promote the consideration of social and economic factors including adequate income through work or pension schemes, social support and social engagement, knowledge of personal health issues, and access to quality healthcare to improve healthy aging. In this Research Topic, we have gathered 28 original articles that examine the relationships between socioeconomic factors, including income, rurality, social support, health literacy, and health expenditure, and the health status of older adults.

The Research Topic report is organized into four broad topic areas with studies from a range of countries, including the United States, China, the Republic of Korea, India, Switzerland, Japan, Northern Ireland, Australia, and Mexico. The groupings are:

- Health Conditions, Health Behaviors, and Health Literacy;
- Loneliness, Social Support, and Social Capital;
- Mental Health and Wellbeing; and
- Service Use, Access, and Expenditure.

The majority of the articles presented secondary analyses of major international aging research datasets including the Survey of Health, Aging and Retirement in Europe (SHARE), the China Health and Retirement Longitudinal Study (CHARLS), Chinese General Social Survey (CGSS), Chinese Longitudinal Healthy Longevity Survey (CLHLS), China Household Finance Survey (CHFS), Korean National Health Insurance (KNHI) database, Korean Community Health Survey (KCHS), Korean Longitudinal Study of Aging (KLoSA), Longitudinal Aging Study in India (LASI), and Northern Ireland Cohort for the Longitudinal Study of Aging (NICOLA). Some cross-sectional studies, a qualitative study, and a commentary are also included. We now review the evidence provided by the articles for each of the four themes.

## Health conditions, multimorbidity, health behaviors, and health literacy

Three studies focused on the prevalence of health conditions and multimorbidity, which increase with age and are higher in low socio-economic status (SES) communities. Nwadiugwu discussed how the management of multimorbidity in older people requires appropriate paradigms and protocols that incorporate the consideration of socio-economic burden and the risk of polypharmacy. Medication use was also explored in Qin S. et al.. Using the 2018 wave from CHARLS, they found that women, those with higher education levels, and participants with poorer self-rated health or multimorbidity were more likely to self-medicate for a self-diagnosed illness. Sinha et al. estimated multimorbidity prevalence at 45.3% in older adults in the lowest economic quintiles using data from Wave 1 of the Longitudinal Aging Study in India (LASI). With each additional condition, personal expenditure increased, as did health insurance claims.

Lifestyle risk factors including smoking, exercise, diet, and sleep quality (3) are important contributors to the onset of health conditions and their management of health as we age. Yuan et al. studied 6,966 Chinese participants aged 60 and above to analyze differences in smoking prevalence and the knowledge, attitudes, and factors associated with smoking among rural and urban participants. The overall prevalence of smoking was 25.6%, with a much higher rate among rural men. The researchers advocated education campaigns focused on older rural men based on their data.

While smoking, diet, and exercise are routinely promoted as key preventive health behaviors, sleep quality is now considered to be an under-estimated key factor affecting the health of older people (4). Zhang M. et al. surveyed 1,189 residents aged 60 and above in China. Measures included self-rated health, sleep quality, mental health, smoking, alcohol, consumption, body mass index, risk of falling, hospitalization, non-communicable diseases, and various socioeconomic measures. Self-rated health was found to be influenced by the number of non-communicable diseases, hospitalization, alcohol consumption, and participant sleep quality. Xue et al. investigated the impact of SES and sleep quality on multimorbidity in a cross-sectional study in Shanxi Province, China. They found, among all SES groups, that older adults with poor sleep quality and low SES had higher multimorbidity. Ayaki et al. studied the onset of wearing presbyopic eyeglasses and their lifestyles among Japanese participants aged between 40 and 59 years. Earlier commencement of near correction was significantly associated with hyperopia, sleeping in, poor subjective sleep quality, and lower annual income. The researchers concluded that healthy sleep may delay the need for near correction and myopia and that sleep disruption might exacerbate presbyopia. These three studies support the importance of sleep quality for the health of older adults.

Health literacy and how we access health information are also important drivers in the prevention and management of health conditions in aging populations (5). Ma et al. examined productive aging in 995 older Chinese adults in a newly urbanized community. They found that education attainment and income had a direct positive effect on health literacy. Based on these findings, they concluded that it would be beneficial to target health education and health promotion for older adults, especially those with a lower SES. Liu et al. studied the impact of frailty among older people in China. They collected data from 637 patients aged 65 and above in Sichuan Province, China, with diabetes and hypertension. To mitigate frailty in this population, they suggested that social support and health literacy education should be provided.

Access to the Internet by older people influences the propensity to access health information and their levels of health literacy (6). Three studies focused on Internet perceptions and use. Guo et al. investigated participants' perceptions of the Internet's importance in obtaining information and the role of learning and social activity in the physical health of older Chinese participating in four waves of the CFPS. The health of women and rural residents and exercise frequency were impacted positively by perceptions of the Internet. Gao et al. examined the perceived importance of the Internet and mobile messaging in life satisfaction as information channels and the self-rated health of older people using two waves of data from the CFPS. Perception of the importance of the Internet positively impacted family atmosphere, life satisfaction, and self-rated health. During the COVID-19 pandemic, online interaction became a more common form of social activity and searching for treatment information. Zhang, Liao et al. conducted a cross-sectional study of 646 people aged 55 and above about their risk perceptions (probability, severity, controllability, and familiarity) during the pandemic and their health information searching behaviors. They found that when facing severe health risks such as COVID-19, older people will search for health information via the Internet.

## Loneliness, social support, and social capital

In previous research, social capital has been linked to better health for older people (Xu Z. et al.). Loneliness and social isolation have been identified as significant issues for older adults impacting on quality of life and risks for dementia, stroke, depression, and anxiety (7). The UN Decade of Healthy Aging (2021–2030) included social isolation and loneliness as key themes.

Bai et al. examined the role of social capital in the self-rated health of older Chinese people living in Anhui Province, surveying 1,810 participants aged 60 and above. Social capital was measured across six dimensions (social participation, social support, social connection, trust, cohesion, and reciprocity). The relationship between social capital and self-rated health (SRH) was not consistent across all the dimensions. Among those with health conditions, poor SRH was associated with lower social participation, cohesion, and reciprocity. Among those with no health conditions, poor SRH was associated with lower social support. Zhou et al. studied the association between social capital and depression among 1,042 older Chinese adults. Reciprocity and social network were significantly negatively correlated with depression among men. Trust, reciprocity, and social participation had a positive effect on reducing depression risk. Among women, social networks and social participation had a negative effect on depression. In the Republic of Korea, Hong et al. studied almost 2 million participants aged 50 and above from the Korean National Health Insurance (KNIHS) database and the Korean Community Health Survey (KCHS). Social capital was measured on one dimension using responses to the question, “Do you trust members of your community?” Dementia was measured using medication data and the ICD-10 classification. Those participants, both men and women, within the highest quintile of trust had the lowest risk of dementia. Comorbidities did not impact this relationship. The authors suggested that trust may support better stress management and hence reduce the risk of dementia.

Wu et al. using data from the SHARE and CHARLS studies, examined the role of income inequality and welfare support in loneliness in Europe and China. They proposed that country income-level inequality may reduce social cohesion and trust, thus increasing loneliness, but providing welfare services and benefits may increase social capital and reduce loneliness. They found that individual socio-economic status (SES) impacted loneliness more in countries with higher income inequality but had a weaker impact in countries with high welfare support. Using data from the CGSS, Qin Y. et al. focused on older Chinese people living alone and found that leisure activities had a positive impact on their physical and mental health but not their social health. Older rural-dwelling people showed lower physical health but higher social health than urban-dwelling people. The authors contended these differences may be driven by lower economic and health service resources and higher neighborhood ties. Burns et al. studied loneliness and its relationship with healthcare use. They analyzed a representative sample of 8,309 community-dwelling adults aged 50 and above from the NICOLA study. They found that the association between loneliness and healthcare usage (HCU) was not significant when health status and health behaviors were controlled.

In a cross-sectional study of 865 participants aged 65 and above in Hubei Province, China, Li et al. examined the relationship between cognition, as measured by the Mini-Mental State Examination (MMSE), and social support, as measured by the Social Support Rating Scale (SSRC). Social support was found to be an important contributor to the quality of life of older people. Using CHARLS, the impact of adult children rural–urban migration on their parents' health was investigated (Zhang, Lyu et al.). Physical health was measured using self-rated health, IADL, and physical activity ability. Other health measures included depression and subjective wellbeing. It was found that the migration of adult children had a positive effect on physical health but not on mental health. The health of fathers but not mothers improved with the migration of adult children.

## Mental health and wellbeing

Four of the Research Topic articles examined social and economic impacts on mental health and wellbeing.

In China, Cheng et al. investigated the impact of residential location on depression using 2018 CHARLS data. The study included 8,210 participants aged 65 and above. Depression was measured using the CES-D, and residential locations were classified as urban (lived in urban areas for 10 years or more), rural–urban (had migrated from rural areas), and rural (lived in rural areas). Depressive symptoms were higher in those who had migrated from rural areas to urban areas. This group often has insufficient income to live well in urban settings with higher living costs. Higher education levels, higher income, and greater engagement in physical activity were associated with lower depressive symptoms. The authors recommended more investment in rural areas to attract people back to these locations and to increase mental health services in urban settings.

Zhao et al. using data from Wave 7 (2018) of the Korean Longitudinal Study of Aging, investigated the relationship between the SES, mental health, and long-term support needs of 5,527 older Koreans aged 60 and above. Mental health was measured using health status satisfaction, quality of life satisfaction, and the CES-D depression scale. The need for long-term care support was measured using ADL (Activities of Daily Living) and IADL (instrumental Activities of Daily Living), activity limitations caused by health issues, hospitalization, and bodily pain. Lower SES and poorer mental health were associated with a higher need for long-term care. The impact of mental health on long-term care needs was higher in men.

Zhang A. et al. analyzed the impact of retirement on happiness for participants in the 2012, 2015, and 2017 waves of the China General Social Survey (CGSS). They found that retirement is associated with increases in men's happiness, but the effects were influenced by level of education (lower education brings greater happiness) and family structure (more children bring greater happiness), and greater health status provided less improvement. The authors argued for a delayed retirement policy.

In an Australian study, Worrall et al. investigated factors associated with depressive symptoms in older adults. Their analyses revealed that life satisfaction, self-esteem, and purpose in life were associated with fewer depressive symptoms. They suggested that depression among older people could be addressed by focusing on enhancing life satisfaction, self-esteem, and purpose in life.

## Service use, access, and expenditure

Ingram et al. investigated potential health inequities in African-American older adults residing in South Carolina, USA. Forty-seven people aged 50–85 years participated in telephone interviews during the early stages of the COVID-19 pandemic. The participants commented on the efficacy and convenience of telehealth consultations, their reactions to the vaccine, its availability, and vaccine hesitancy, as well as the need for more engaged physicians and family support. The authors concluded that more qualitative lived experience research is needed.

Bachmann et al. studied 60,209 Swiss community-dwelling patients aged 75 and above who had been hospitalized with a chronic health condition. They modeled the impact of social and regional factors on the odds of a nursing home admission after hospital discharge, which occurred for 7.8% of all patients. They found that higher education level, insurance status, and living alone altered the chance of being admitted to a nursing home. They suggested that social care and healthcare could work together to reduce institutionalization rates.

The impact of population aging on health expenditure has been hotly debated globally (8). Many health conditions are age related, and health spending is driven by chronic illnesses such as heart disease and mental health conditions (9). In its latest report, the OECD highlights the increase in expenditure on health by governments in response to the COVID-19 pandemic (10). Using data from the China Household Finance Survey (CHFS), Xu X. et al. concluded that age, income, family size, self-rated health, and the old-age dependency ratio influenced household health expenditure in China, with greater impact in eastern China.

Salinas-Rodríguez et al. studied out-of-pocket (OOP) healthcare costs for an older sample of 735 community-dwelling Mexican adults aged 60 and above with IADL and ADL dependence. They found that ADL and IADL dependence are highly influential for annualized OOP healthcare expenditures. Their findings highlighted the economic implications of the dependence on individuals, their families, and the health system in Mexico and the importance of policies to address them.

## Conclusion

While articles from China were strongly represented, this Research Topic incorporated studies from a wide range of countries. A strength of this collection is the inclusion of analyses of high-quality gold standard longitudinal studies. However, greater commonality in and standardization of measurement tools and domains would permit better quality cross-country comparisons. There was limited treatment of urban–regional differences in health outcomes among the studies. Rural populations are often disadvantaged in terms of health access and health outcomes (11). The Research Topic included articles with inconsistent application of multimorbidity and disease burden concepts, which are critical for addressing the UN Sustainable Development Goal of reducing premature death due to non-communicable diseases (12). Our submissions did not take advantage of the strengths of using harmonized datasets from the Gateway to Global Aging Data initiative, which permits cross-country comparisons of core content areas.^1^ In a recent article, we have argued for the use of health economics concepts and interdisciplinary co-operation, including biomedical and psychosocial markers, to transform approaches to chronic disease prevention and management across the lifespan (3). We repeat that call in this editorial. An integrated approach utilizing health economics concepts and based on high-quality, large-scale data collections will deliver the best quality and usable results to inform health and social policies and outcomes.

## Author contributions

CB: Conceptualization, Writing—original draft, Writing—review & editing. KL: Writing—review & editing. ST: Conceptualization, Writing—original draft, Writing—review & editing.

## Footnotes

^1^Gateway to Global Aging Data. Available online at: https://g2aging.org/survey-overview.
